# Hemophagocytic lymphohistiocytosis and congenital factor VII deficiency: a case report

**DOI:** 10.1186/s12881-018-0673-y

**Published:** 2018-09-12

**Authors:** Xiong Wang, Ning Tang, Wei Chang, Yanjun Lu, Dengju Li

**Affiliations:** 1 Department of Laboratory Medicine, Tongji Hospital, Tongji Medical College, Huazhong University of Science and Technology, Wuhan, 430030 China; 2 Department of Hematology, China Resource & WISCO General Hospital, Wuhan University of Science and Technology, Wuhan, 430081 China; 3 Department of Hematology, Tongji Hospital, Tongji Medical College, Huazhong University of Science and Technology, Wuhan, 430030 China

**Keywords:** Hemophagocytic lymfohistiocytosis, Infection, Hemophagocytosis, Factor VII deficiency

## Abstract

**Background:**

Hemophagocytic lymfohistiocytosis (HLH) is a rare, life-threatening hyperinflammation, characterized by immune system over-activation resulting in hemophagocytosis. HLH could appear as a primary disease caused by mutations of immune-regulatory genes, or develop as a result of viral or bacterial infections, or malignancy. Congenital factor VII (FVII) deficiency is a rare autosomal recessive disorder characterized by prolonged prothrombin time (PT) and low FVII, which may increase bleeding risk.

**Case presentation:**

A 50-year-old woman was admitted for a fever persisted for 20 days, presenting with cytopenia, high hyperferritinemia, low activity of NK cells. Bone marrow aspiration showed hemophagocytosis. CT scanning found pulmonary infection. EBV and CMV were not detected. Genetic scanning did not find pathogenic mutation of a HLH NGS panel including 26 genes. This patient was treated as recommended by the HLH 2004 Guidelines. Coagulation tests identified FVII deficiency. Genetic analysis of *F7* gene in the patient and her family members identified recurrent compound heterozygous *F7* c.64 + 5G > A and c.1224 T > G (p.His408Gln) mutations in this patient and her brother who showed postoperative hemorrhage after surgical resection of renal cell carcinoma. Heterozygotes in this family were asymptomatic.

**Conclusions:**

To our knowledge, this is the first report of HLH in combination with congenital FVII deficiency in Chinese population.

## Background

Hemophagocytic lymphohistiocytosis (HLH) is a very rare life-threatening syndrome characterized by excessive immune activation and hyperinflammation [1]. HLH can be either familial or secondary to infection, immunosuppression, autoimmune disease, and malignancy [2]. Acquired HLH is an aggressive clinical entity which require early diagnosis and appropriate therapies. Management of HLH was indicated in the HLH-2004 guidelines, although it was heavily debated [3]. An especially high incidence of Epstein-Barr virus (EBV)-associated HLH (EBV-HLH) was found in Asia [4, 5].

Congenital factor VII (FVII) deficiency (OMIM: 227500) is a rare inheritable coagulation disorder with an estimated prevalence of 1:500000, and is inherited in autosomal recessive (AR) model. Clinical manifestations vary from asymptomatic to severe bleeding, and the clinical manifestations do not correlate well with plasma FVII levels, which lead to the patient management during surgery challenging [6]. Congenital FVII deficiency is characterized by prolonged prothrombin time (PT) and low FVII. 30% was considered as the cutoff for clinical manifestations in patients with FVII deficiency [7].

We hereby report the first case of patient suffered from both HLH and congenital FVII deficiency.

## Case presentation

### HLH

The 50-year-old woman was admitted for a fever persisted for 20 days. Computed tomography (CT) scanning showed pulmonary infection. Cytopenia was observed in peripheral blood. White blood cells, red blood cells, and neutrophil graneulocytes were all decreased. Hemoglobin was only 74.0 g/L. Ferritin was increased to be 3602.5 g/L. Autoimmune antibody test found no abnormity. The available laboratory data were summarized in Table 1.

**Table 1:** Laboratory test results

Test	Result	Reference	Unit
WBC	1.24 (↓)	3.50–9.50	10^9^/L
GRA (%)	61.3	40.0–75.0	%
GRA (#)	0.76 (↓)	1.80–6.30	10^9^/L
LYN (%)	37.9	20.0–50.0	%
LYN (#)	0.47 (↓)	1.10–3.20	10^9^/L
MONO (%)	0.8 (↓)	3.0–10.0	%
MONO (#)	0.01 (↓)	0.10–0.60	10^9^/L
EOS (%)	0.0 (↓)	0.4–8.0	%
EOS (#)	0.00 (↓)	0.02–0.52	10^9^/L
BAS (%)	0.0 (↓)	0–1.0	%
BAS (#)	0.00 (↓)	0.00–0.06	10^9^/L
RBC	2.67 (↓)	3.80–5.10	10^12^/L
HB	74.0 (↓)	115.0–150.0	g/L
HCT	22.0 (↓)	35.0–45.0	%
MCV	82.4	82.0–100.0	fL
MCH	27.7	27.0–34.0	pg
MCHC	336	316–354	g/L
PLT	146.0	125.0–350.0	10^9^/L
PDW	10.3	9.0–17.0	fL
MPV	9.9	8.0–15.0	fL
P-LCR	23.3	13.0–43.0	%
PCT	0.14	0.10–0.25	%
FER	3602.5 (↑)	15–150	μg/L
ALT	53 (↑)	≤33	U/L
AST	60 (↑)	≤32	U/L
LDH	737 (↑)	135–214	U/L
ALP	126 (↑)	35–105	U/L
EBV	Negative	Negative	–
CMV	Negative	Negative	–

*WBC* white blood cell, *GRA* neutrophil graneulocyte, *LYN* lymphocyte, *MONO* Monocyte, *EOS* eosinophil, *BAS* basophil, *RBC* red blood cell, *HB* hemoglobin, *HCT* hematocrit, *MCV* erythrocyte mean corpuscular volume, *MCH* mean corpuscular hemoglobin, *MCHC* mean red blood cell hemoglobin concentration, *PLT* platelet, *PDW* platelet distribution width, *MPV* mean platelet volume, *P-LCR* proportion of large platelet, *PCT* plateletcrit, *FER* ferritin, *ALT* alanine aminotransferase, *AST* aspartate aminotransferase, *LDH* lactate dehydrogenase, *ALP* alkaline phosphatase, *EBV* Epstein-Barr virus, *CMV* Cytomegalovirus

**Fig. 1:**
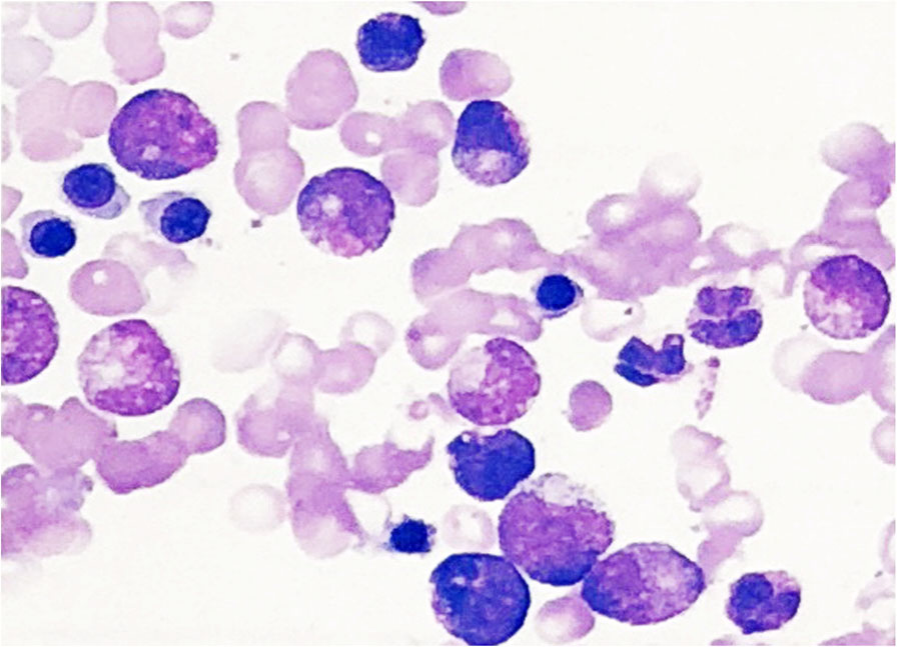
Bone marrow aspiration. Mononuclear histiocyte with engulfed erythrocyte was observed. G = 64.5%, E = 31.5%, G/E = 2.05:1

NK cells activity was detected according to IFN-γ secretion by using whole blood as previously established in our laboratory [8]. Moreover, functional activity of NK cells was detected using K562 cells as target cells. Low activity of NK cells was found in two assays. In both assays, the activity of NK cells was only 30% of the low limit of healthy controls. Bone marrow aspiration confirmed hemophagocytosis (Fig. 1). Laboratory tests exclude EBV or Cytomegalovirus (CMV) infection, common cause of HLH. This patient was diagnosed and treated according to HLH-2004 guidelines [9, 10].

To explore the genetic cause of HLH in this patient, a targeted next generation sequencing (NGS) panel was applied, including *LYST, CTPS1, PIK3CD, PRF1, SRGN, CD27, LAMP1, ARF6, GZMB, RAB27A, BLOC1S6, CORO1A, UNC13D, STXBP2, GNLY, STK4, PRKCD, AP3B1, ITK, STX11, CARD11, MCM4, MAGT1, SH2D1A, XIAP,* and *IL2RG* genes. The mean depth was 315 folds. 98.44% of target region was covered by at least 20 folds. The NGS was performed on the Ion Torrent Personal Genome Machine as previously described [11]. However, NGS targeting HLH associated gene found no pathogenic variant.

### Congenital FVII deficiency

**Fig. 2:**
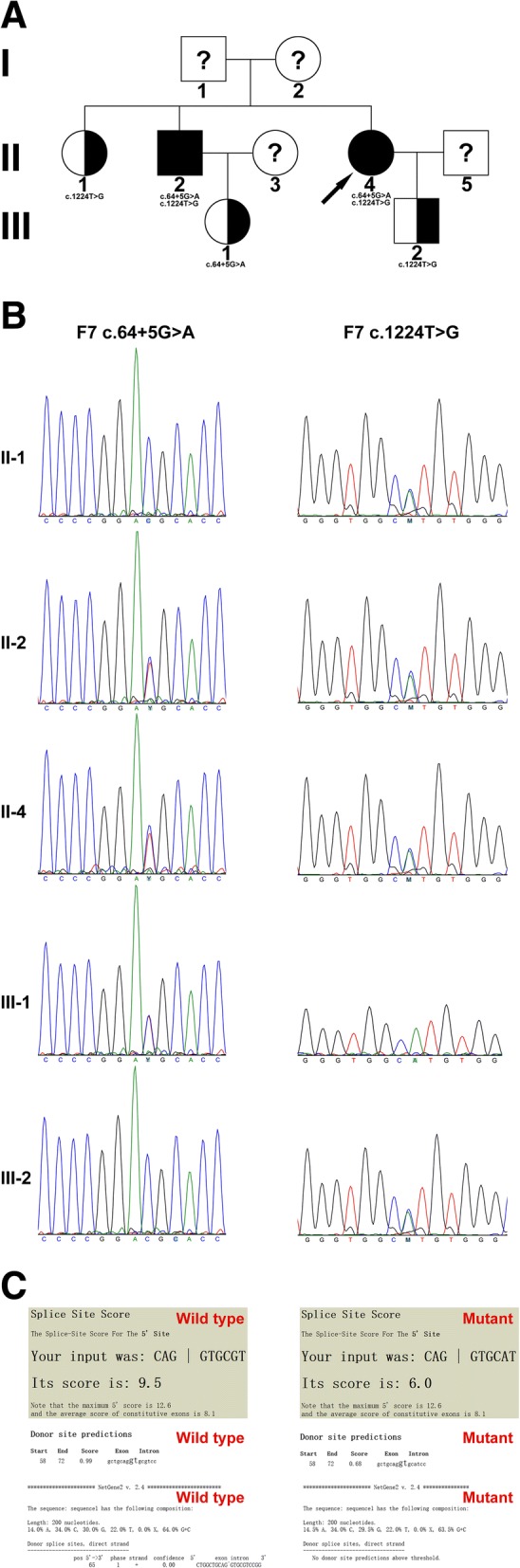
Congenital factor VII (FVII) deficiency. **a**, The family tree of a Chinese family with HLH and congenital FVII deficiency. Square and circle denoted male or female respectively. Full-filled square and circle meant patients, and half-filled symbols represented heterozygous carrier. The arrow indicated the proband. A question mark meant that genetic analysis was unavailable. **b**, Sanger sequencing of *F7* c.64 + 5G > A and c.1224 T > G mutations. **c**, Splicing site prediction by Splice Site Score Calculation (http://rulai.cshl.edu/new_alt_exon_db2/HTML/score.html), Splice Site Prediction by Neural Network (http://www.fruitfly.org/seq_tools/splice.html), and Netgene2 (http://www.cbs.dtu.dk/services/NetGene2/)

Coagulation tests showed the FVII:C was decreased to be 4%. The FVII:C of the patient’s brother was 5%, who suffered postoperative hemorrhage after surgical resection of renal cell carcinoma 3 years ago. Both the patient and her brother showed prolonged PT. Family tree was drawn (Fig. 2a). Genomic DNA was extracted from peripheral blood mononuclear cell (PBMC). Coding exons and adjacent splice junctions were amplified for the *F7* gene. Sanger sequencing was performed bi-directionally on ABI 3500 Dx. NM_000131.4 was used as reference transcript of the *F7* gene. Genetic analysis of the *F7* gene in the patient and her family members identified recurrent compound heterozygous *F7* c.64 + 5G > A and c.1224 T > G (p.His408Gln) mutations in this patient and her brother. Heterozygotes were found in other family members who showed slightly decreased FVII:C (Fig. 2b, Table 2). Heterozygotes were asymptomatic.

**Table 2:** Congenital FVII deficiency

No.	Age (year)	F7 c.64 + 5G > A	F7 c.1224 T > G	PT	APTT	FVII:C
II-1	63	Wild type	Heterozygous	14.2	–	67
II-2	61	Heterozygous	Heterozygous	28.4	38.9	5
II-4	50	Heterozygous	Heterozygous	30.3	34.6	4
III-1	36	Heterozygous	Wild type	14.4	33.5	45
III-2	28	Wild type	Heterozygous	14.5	37.1	51

## Discussion and conclusions

HLH is a severe or fatal inflammatory condition caused by hereditary or acquired immunoregulatory abnormity. Inflammatory cytokine storm caused by the excessive activation and proliferation of macrophages and T-lymphocytes may contribute to HLH pathology [12, 13]. Primary HLH is an autosomal or X-linked recessive immune disorders. Secondary HLH is often precipitated by infection, autoimmune disease, malignancy, or metabolic conditions, and its prognosis is poor. The initial clinical manifestations of HLH may vary widely and lead to misdiagnosis. For secondary HLH, 8 criteria were proposed (fever, splenomegaly, cytopenia, hypertryglyceridemia or hypofibrinogenemia, high ferritin, elevated soluble CD25, low NK cell activity, and hemophagocytosis in biopsy) and the presence of 5/8 of these criteria confirmed the diagnosis [10]. Prompt start of therapy was essential and lifesaving. In this study, the patient received anti-infection therapy with Moxifloxacin Hydrochloride for 6 days, and then she was transferred to our department and treated according to HLH-2004 when her fever persisted for 20 days. CT scanning showed pulmonary infection. EBV was not detected, and malignancy could not be excluded.

FVII is involved in the ‘initiation’ phase via binding tissue factor exposed by cells after endothelial injury. The complex promotes the activation of factor X and IX, leading to the generation of thrombin [14]. Congenital FVII deficiency is an AR disorder, which is defined by the complete absence or below 70% of normal of FVII [15]. Clinical manifestations of FVII deficiency were heterogeneous, varying from asymptomatic to fatal bleeding, which do not correlate well with FVII plasma levels. Laboratory test for FVII activity is the first-line method for FVII deficiency diagnosis.

In this family, compound heterozygous *F7* c.64 + 5G > A and c.1224 T > G mutations were found in the patient and her brother. Homozygous *F7* c.64 + 5G > A mutation has been previously reported [16]. Peyvandi F et al., reported that this mutation might result in the preservation of some FVII coagulant activity and was associated with a mild bleeding history. Three kinds of splicing software were used to predict the effect of *F7* c.64 + 5G > A mutation on 5′ splicing site (Fig. 2c). The 5′ splicing site of exon 1 was predicted to be greatly influenced by *F7* c.64 + 5G > A mutation. *F7* c.1224 T > G mutation resulted in amino acid substitution of His^408^ to Gln. Katsumi A et al., reported that his mutation leads to impaired secretion of the molecule and FVII deficiency in vitro [17]. In this family, heterozygotes showed slightly decreased FVII:C, while the patient and her brother showed greatly decreased FVII:C level, both of whom carried compound heterozygous *F7* c.64 + 5G > A and c.1224 T > G mutations. These results were consistent with the AR inheritance model.

Bleeding and altered coagulation can occur in patients with HLH, but, vice versa, coagulation defects are associated to more severe HLH [18, 19]. The most frequently reported hemostasis abnormity is hypofibrinogenemia partially due to fibrinogen consumption by disseminated intravascular coagulation (DIC) [1]. DIC and thrombocytopenia were associated with adverse outcome in HLH patients [20]. FVII interacts with tissue factor and activates factor X (FX) binding to platelets, leading to thrombin formation. Thrombin plays a role in the activation of platelets, cleavage of fibrinogen to produce fibrin, and stabilization of clot by the actions of activated factor XIII [21]. Recombinant factor VIIa (rFVIIa) has been used successfully in HLH patients with severe hemorrhage [22, 23]. FVII deficiency may increase the bleeding risk of HLH patients.

## Abbreviations

AR: Autosomal recessive
CMV: Cytomegalovirus
CT: Computed tomography
EBV: Epstein-Barr virus
FVII: Factor VII
HLH: Hemophagocytic lymphohistiocytosis
NGS: Next generation sequencing
PBMC: Peripheral blood mononuclear cell
PT: Prothrombin time
